# Mechanistic determinants of the directionality and energetics of active export by a heterodimeric ABC transporter

**DOI:** 10.1038/ncomms6419

**Published:** 2014-11-07

**Authors:** Nina Grossmann, Ahmet S. Vakkasoglu, Sabine Hulpke, Rupert Abele, Rachelle Gaudet, Robert Tampé

**Affiliations:** 1 Institute of Biochemistry, Biocenter, Goethe-University Frankfurt, Max-von-Laue-Street 9, D-60438 Frankfurt/M., Germany; 2 Department of Molecular and Cellular Biology, Harvard University, 52 Oxford Street, Cambridge, Massachusetts 02138, USA; 3 Cluster of Excellence Frankfurt—Macromolecular Complexes, Goethe-University Frankfurt, Max-von-Laue-Street 9, D-60438 Frankfurt/M., Germany

## Abstract

The ATP-binding cassette (ABC) transporter associated with antigen processing (TAP) participates in immune surveillance by moving proteasomal products into the endoplasmic reticulum (ER) lumen for major histocompatibility complex class I loading and cell surface presentation to cytotoxic T cells. Here we delineate the mechanistic basis for antigen translocation. Notably, TAP works as a molecular diode, translocating peptide substrates against the gradient in a strict unidirectional way. We reveal the importance of the D-loop at the dimer interface of the two nucleotide-binding domains (NBDs) in coupling substrate translocation with ATP hydrolysis and defining transport vectoriality. Substitution of the conserved aspartate, which coordinates the ATP-binding site, decreases NBD dimerization affinity and turns the unidirectional primary active pump into a passive bidirectional nucleotide-gated facilitator. Thus, ATP hydrolysis is not required for translocation *per se*, but is essential for both active and unidirectional transport. Our data provide detailed mechanistic insight into how heterodimeric ABC exporters operate.

## Abstract (web summary)

ABC systems are an important class of transporters involved in human pathologies for which the basis for transport remains poorly understood. Here the authors suggest a molecular basis for ATP-powered vectorial peptide export by the human transporter associated with antigen processing (TAP).

## Graphical abstract

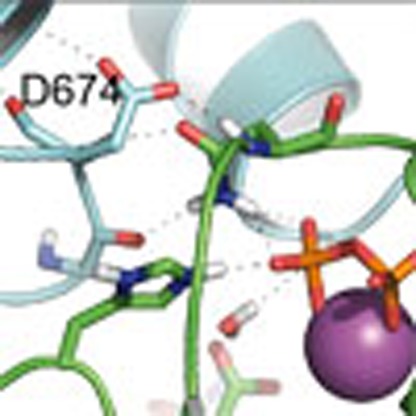

Life depends on transport systems that shuttle ions and molecules across cell membranes. ATP-binding cassette (ABC) transporters, which represent the largest family of primary active transporters, convert the energy of ATP binding and hydrolysis via two NBDs into conformational changes of two transmembrane domains and thus move a wide range of essential as well as harmful substrates across membranes^1^,^2^,^3^,^4^,^5^. In humans, ABC transporters participate in a wide range of important cellular processes such as chloride homeostasis, cholesterol and lipid metabolism, insulin secretion, detoxification and adaptive immunity. Accordingly, ABC transporter malfunction is linked to many pathological conditions, including cystic fibrosis, diabetes, adrenoleukodystrophy, multidrug resistance, neurological and infectious diseases^6^.

The ABC transporter associated with antigen processing (TAP) plays a prime role in adaptive immunity by supplying proteasomal degradation products for loading of major histocompatibility (MHC) class I molecules in the ER lumen. This sophisticated peptide loading process is accomplished by a macromolecular complex composed of TAP, tapasin, calreticulin, ERp57 and MHC I (refs 7, 8, 9). Loaded MHC I molecules then traffic to the cell surface to present their antigenic cargo to CD8^+^ cytotoxic T cells for immune surveillance, detection and elimination of abnormalities such as virally or malignantly transformed cells.

TAP consists of two ABC half-transporters, TAP1 and TAP2, each composed of a transmembrane domain (TMD) followed by an NBD^5^. The two NBDs are thought to run through a cycle of dimer formation and dissociation controlled by ATP binding and hydrolysis, respectively, which in turn drives the TMDs from an inward- to an outward-facing conformation and back^10^,^11^,^12^. The communication between the TMDs and NBDs is mediated by interactions between two coupling helices found in each TMD and conserved regions of the NBDs^10^,^13^. The NBDs contain conserved motifs with distinct functions in ATP binding and hydrolysis. Interestingly, the two composite ATP-binding sites are functionally non-equivalent. The Walker A and B motifs of TAP2 and the C-loop of TAP1 form the active ATP-binding site II (Fig. 1a), whereas the degenerate ATP-binding site I displays a very low ATPase activity^14^,^15^,^16^,^17^.

The D-loop is the second hallmark found in all ABC transporters, besides the signature C-loop essential for ATP hydrolysis. However, the function of this conserved ‘SALD’ sequence motif is not well characterized and contradictory results exist. In SUR1, substitution of the aspartate by a cysteine in one of the NBDs interferes with the gating of the associated Kir6.2 channel^18^. On the other hand, exchange of the D-loop aspartate to alanine in the sterol transporter ABCG5/8 had no impact on sterol transport *in vivo*^19^. In the homodimeric lipid A exporter MsbA, substitution of the conserved aspartate to glycine strongly interferes with bacterial viability^20^. In ATP-bound NBD dimers, the two D-loops contact one another at the dimer interface (Fig. 1a) and position the Walker A motif and H-loop of the opposite NBD via a complex hydrogen bond and electrostatic network^21^,^22^. Moreover, the D-loop and Walker B motifs position, coordinate and activate the putative attacking water for ATP hydrolysis^17^,^23^. By forming contacts with the ATP-binding sites in *cis* and *trans*, the D-loops are in a prime position to mediate communication between both ATPase sites.

Despite the important task of TAP in immune surveillance, fundamental questions regarding antigen translocation by TAP, questions that are also relevant to other ABC export systems, remain to be answered: (i) Can the TAP complex pump peptide substrates against a concentration gradient? (ii) Does TAP operate uni- or bidirectionally? (iii) What determines its directionality? Previous studies in semi-permeabilized cells or isolated microsomes relied on the trapping of translocated peptides into the ER lumen by glycosylation to prevent export from the ER^24^, making it impossible to tackle these questions. Moreover, determining the concentration of free peptide in the ER lumen is important to understand peptide editing and selection of immunodominant epitopes. These questions are also difficult to address in other well-studied ABC transporters, many of which transport hydrophobic substrates that partition to some extent within the lipid bilayer. To the best of our knowledge, the only report of substrate accumulation against a concentration gradient for an ABC exporter is that of P-gp (ABCB1), where tenfold substrate accumulation has been estimated^25^. Furthermore, the directionality of transport was challenged by the observation that a bacterial homologue of P-gp may function as ATP synthase at the expense of drug transport in the reverse direction^26^.

To address these questions, fundamental to the function of primary transporters, we reconstituted TAP in proteoliposomes and performed influx and efflux assays. We demonstrate that TAP accumulates peptides in a unidirectional manner to a threshold concentration determined by trans-inhibition. Transport unidirectionality and the generation of a concentration gradient are coupled to ATP hydrolysis as we have identified a specific D-loop mutant inactive in ATP hydrolysis, which functions as a nucleotide-gated facilitator. Furthermore, this mutation has only a minor impact on ATP and peptide binding. Using rat TAP1 NBD homodimerization as a model system for biophysical measurements and structural studies, we showed that the D-loop mutation decreases the ATP-driven NBD dimerization affinity by nearly an order of magnitude and affects NBD dimer structure in subtle ways. We combine our biochemical, biophysical and structural data to propose a model in which peptide release to the lumenal side is a checkpoint for ATP hydrolysis, thus making ATP hydrolysis the key step in unidirectional peptide transport.

## Results

### D-loop mutation enables translocation without ATP hydrolysis

**Figure 1:**
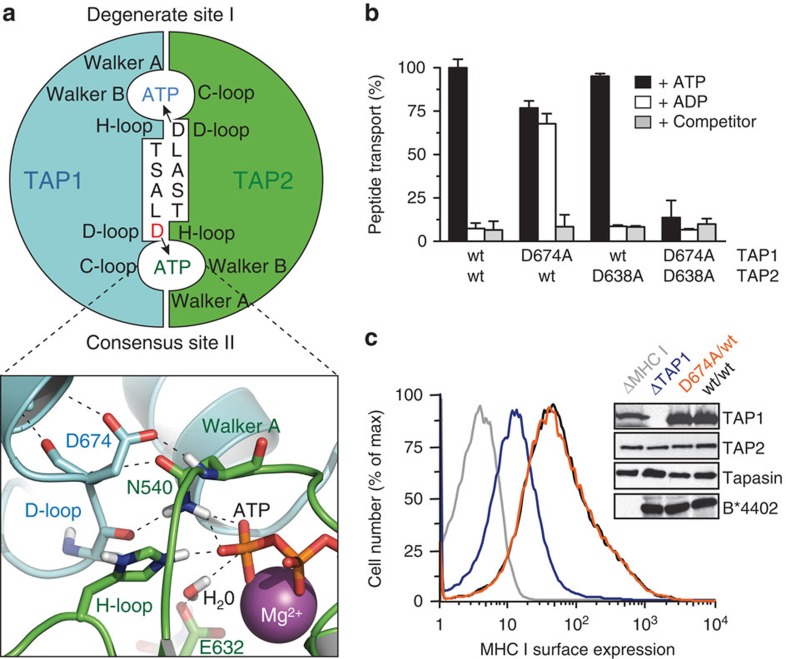
A D-loop mutation in TAP1 enables antigen peptide export without ATP hydrolysis. (**a**) Two asymmetric ATP-binding sites are formed at the NBD dimer interface of TAP1 (cyan) and TAP2 (green). The conserved aspartate in each D-loop contributes to the degenerate site I and consensus site II formed by the Walker A and B of the opposite subunit. The NBD interface is modelled on the TAP1-NBD dimer (PDB 2IXE)^17^. The electrostatic and hydrogen bond network of the active site II is enlarged in the lower panel. Key amino-acid residues, ATP and Mg^2+^ (sphere) are illustrated. (**b**) Transport of fluorescein-labelled peptide RRYQNSTC^(FL488)^L (1 μM) across ER membranes containing equal amount of wt TAP and D-loop variants was analysed in the presence of ATP, ADP (3 mM each) or 200-fold molar excess of unlabelled competitor peptide plus ATP for 3 min. Transported peptides were quantified fluorometrically. Data are normalized to wt TAP and presented as means±s.d. (*n*=3). Equal expression levels of TAP1 and TAP2 were confirmed by immunoblotting. (**c**) MHC class I antigen processing was established by expressing wt TAP (black) or the D674A/wt complex (orange) in the presence of tapasin and MHC I (HLA-B*4402). MHC I surface expression was monitored by flow cytometry using the conformation-specific anti-HLA-A/B/C antibody W6/32. The absence of TAP1 (blue) or MHC I (grey) resulted in a suppression of MHC I surface expression or background level, respectively.

We focused our mechanistic studies on the D-loops of TAP, whose functions are ill-defined. By scanning mutagenesis, we analysed first the D-loop of TAP1, which forms part of the active ATP-binding site II (Supplementary Fig. 1). Similar to wild-type (wt) TAP, all D-loop mutants showed ATP-dependent peptide transport activity (Supplementary Fig. 2a). Surprisingly, the D674A/wt complex translocates peptides not only in the presence of ATP but also of ADP (Fig. 1b). ADP binds to TAP with a similar affinity to that of ATP but cannot energize peptide transport of wt TAP^27^,^28^,^29^. Interestingly, the corresponding D-to-A mutation in TAP2 (wt/D638A), affecting the degenerate ATP-binding site I, behaves like wt TAP. In contrast, the double D-loop mutant D674A/D638A is inactive in peptide transport. It is worth noting that all mutants displayed similar affinity for peptides and ATP as compared with wt TAP (Supplementary Table 1).

We also addressed the physiological consequences of the surprising D-loop substitution. To this end, the pathway of MHC I antigen processing was reconstituted in insect cells lacking the components of this pathway. The cell surface expression of the MHC I allele B*4402 was monitored by flow cytometry (Fig. 1c). Expression of wt TAP and D674A/wt mutant leads to a similar upshift in MHC I cell surface expression, whereas the lack of TAP1 impairs MHC I antigen presentation. These results indicate that the energetically uncoupled D674A/wt complex promotes antigen processing and MHC I presentation to the same extent as wt TAP.

### D-loop enables coupling of transport and hydrolysis

**Figure 2:**
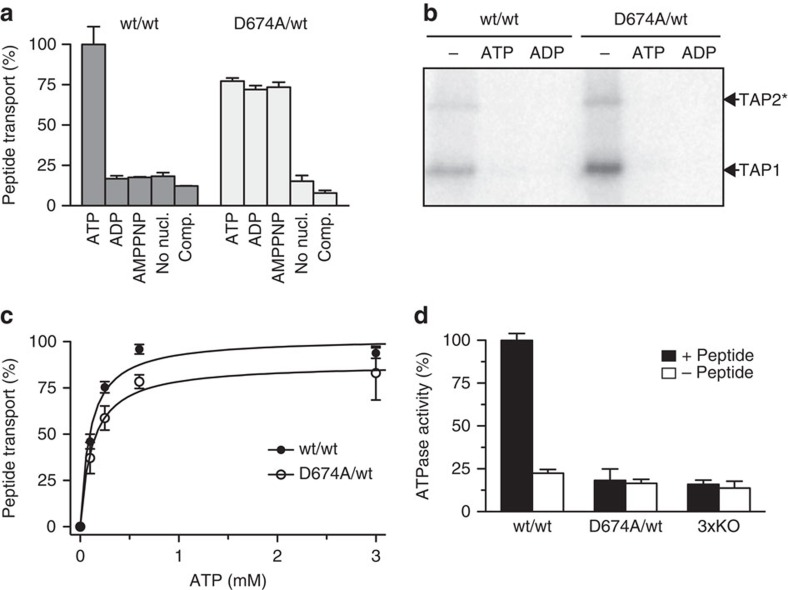
The D-loop controls the allosteric coupling between peptide transport and ATP hydrolysis. (**a**) Transport of fluorescein-labelled peptide RRYC^(FL488)^KSTEL (1 μM) by reconstituted TAP (1.1 μg initial amount) was analysed for 10 min in the presence of different nucleotides (3 mM each) or ATP in the presence of a 200-fold excess of unlabelled peptide (comp.). (**b**) Purified wt TAP or the D674A/wt complex (0.5 μM each) was incubated with 8-azido-[α-^32^P]-ATP (1 μM) in the absence or presence of a 200-fold excess of ATP or ADP. Cross-linked products were separated by SDS–PAGE (6%) and visualized by autoradiography. *Tapasin-TAP2 fusion used to separate the TAP subunits^40^. (**c**) ATP-dependent peptide transport was analysed in the presence of RRYC^(FL488)^KSTEL (1 μM). (**d**) Hydrolysis of ATP (3 mM) by purified TAP variants (0.5 μM each) was analysed for 20 min in the presence (black) or absence (white) of peptide RRYQKSTEL (1 μM). An ATPase inactive TAP complex (3xKO) with a triple mutation in the Walker A of TAP1 and TAP2 (K544A and K509A, respectively) and H-loop of TAP2 (H661A) was used as control to measure TAP-independent residual ATPase activity^30^. Data are normalized to wt TAP and presented as means±s.d. (*n*=3).

To explore the molecular mechanism of antigen translocation, wt TAP and the D674A/wt complex were purified and functionally reconstituted in proteoliposomes. Peptide translocation by wt TAP is strictly ATP dependent, whereas the D674A/wt TAP mediated peptide translocation even in the presence of ADP or non-hydrolysable adenosine 5′-(β,γ-imido)triphosphate (AMPPNP) (Fig. 2a). Notably, both wt TAP and D674A/wt need Mg^2+^ ions for peptide translocation (Supplementary Fig. 2b). Also, wt and D674A/wt TAP showed the same binding properties for ATP, ADP (Fig. 2b) and peptides (Supplementary Table 1). Consistent with these data on full-length human TAP, a fluorescence polarization-based competition assay showed that the affinities for ATP and ADP of isolated wt and D-loop (D651A) mutant rat TAP1-NBD are similar (Supplementary Table 2). Furthermore, the Michaelis–Menten kinetics for peptide transport by wt TAP and D674A/wt are equal (Fig. 2c, Supplementary Fig. 2c and Supplementary Table 3), confirming that the affinity for ATP and peptides is not affected by the D-loop substitution.

We next analysed the coupling between peptide translocation and ATP hydrolysis. As shown in Fig. 2d, peptide-stimulated ATP hydrolysis by wt TAP was observed. Notably, TAP has no basal ATPase activity as concluded from a comparison with an ATPase inactive mutant (3xKO)^30^. In contrast to wt TAP, the D674A/wt complex is inactive in ATP hydrolysis, either in the presence or absence of peptide substrate. Consistently, mutating the D-loop of the isolated rat TAP1-NBD also impairs its ATPase activity (Supplementary Fig. 3). Therefore, peptide transport still occurs despite impairments in ATP hydrolysis, indicating that the crosstalk between peptide translocation and ATP hydrolysis is disrupted.

### Active exporter turned into a nucleotide-gated facilitator

**Figure 3:**
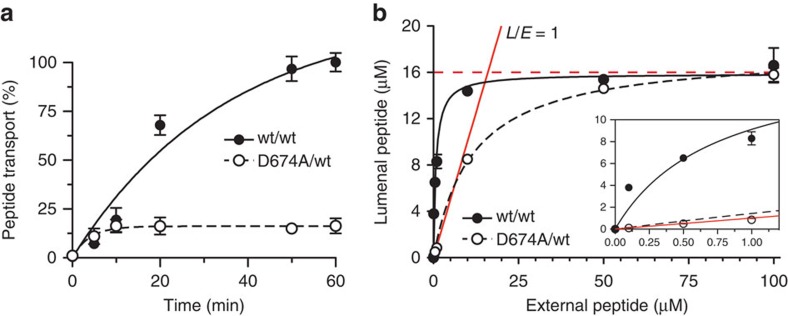
TAP is an active peptide export pump, whereas D674/wt is a facilitator. (**a**) Time-dependent peptide transport of reconstituted wt TAP and D674A/wt complex (1.1 μg initial amount each) was followed in the presence of peptide RRYC^(FL488)^KSTEL (1 μM) and ATP (3 mM). Data are normalized to the transported peptide by wt TAP after 1 h. (**b**) The concentration of peptides accumulated by wt or D674A/wt TAP was analysed after 1 h at different concentrations of external Atto633-labelled peptide RRYC^(AT633)^KSTEL. The internal volume of the liposomes was quantified by encapsulated 5(6)-carboxyfluorescein. The red line reflects a lumenal-to-external peptide ratio (L/E) of 1 (passive flux). The dashed red line illustrates trans-inhibition at ~16 μM. The inset shows the curves and fit in the low peptide concentration range.

We then examined the kinetics and energetics of antigen translocation. Over time, wt TAP transports peptides to much higher levels than the D674A/wt complex (Fig. 3a). However, the initial translocation rate constants (zero-trans flux) of wt TAP and D674A/wt were comparable at 11.1±1.0 and 10.1±0.8 nmol min^−1^ mg^−1^, respectively, implying that the rate of conversion from the inward- to the outward-facing conformation does not depend on ATP hydrolysis. Unfortunately, loss of TAP activity prevented observations over incubation times longer than 1 h. Quantification of the lumenal concentration revealed that if 1 μM of peptide was added externally, wt TAP accumulated peptides to 8.3±0.6 μM, whereas D674A/wt reached 0.9±0.3 μM (Fig. 3b). At low concentration of peptides added (0.1 μM), a 38-fold accumulation was observed for wt TAP, whereas the D674A/wt complex equilibrated at 0.1 μM. These experiments prove for the first time that TAP uses the chemical energy of ATP to pump peptides against the concentration gradient. In stark contrast, the D-loop mutation disrupts the tight coupling between peptide translocation and ATP hydrolysis, allowing only for a passive peptide flux, which still requires binding of ADP or ATP.

### Trans-inhibition at high lumenal substrate concentration

Notably, at high substrate concentrations, for example, 100 μM, the translocated peptides did not reach above 16 μM in the liposomes for wt TAP and D674A/wt, ruling out that the D-loop mutation converts the transporter into a channel. Furthermore, these results are not affected by the amount of TAP reconstituted per liposome (Supplementary Fig. 4). Also, single vesicle-based translocation assays show that more than 95% of the proteoliposomes are transport active, establishing that each liposome contains at least one active transport complex. An initial lipid-to-TAP (w/w) reconstitution ratio of 20 leads to approximately 300,000 lipids per active and correctly oriented TAP complex, ensuring that peptide accumulation is not underestimated by low reconstitution efficiency (Supplementary Fig. 5). These data indicate that TAP is inhibited in *trans* by accumulated lumenal peptide. Because of active transport by wt TAP, trans-inhibition is reached at a much lower external peptide concentration than for the D-loop mutant. Because substrate translocation in the D674A/wt complex is uncoupled from ATP hydrolysis, the measured EC_50_ can be attributed to a low-affinity peptide-binding site, exposed to the vesicle lumen, with a *K*_d_ of 10.7±0.5 μM. It is worth noting that peptide transport is not inhibited by proton or ion gradients, because nigericin or valinomycin did not affect the transport activity either of wt TAP or D674A/wt (Supplementary Fig. 6). These results provide direct evidence that TAP operates as primary active transporter, which is inhibited by saturating a low-affinity binding site that is accessible from the ER lumen. We propose that this trans-inhibition process is important for a fine-tuned balance between antigen processing and ER homeostasis, thereby preventing a harmful accumulation of peptides in the ER and induction of ER stress^31^.

### ATP hydrolysis mediates unidirectional/vectorial transport

**Figure 4:**
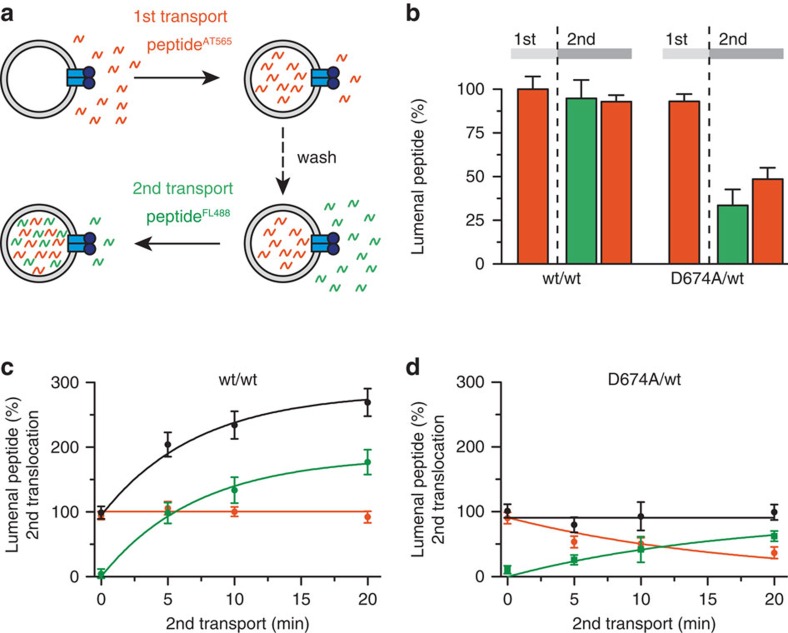
The D-loop sets the directionality of antigen translocation. (**a**) Vectoriality of antigen translocation by TAP was demonstrated by dual-colour transport assays with Atto565 (red)- and fluorescein (green)-labelled peptides (RRYQNSTC^(AT565)^L and RRYQNSTC^(FL488)^L, respectively). (**b**–**d**) After the first 10-min translocation of RRYQNSTC^(AT565)^L (1 μM; initial TAP amount for reconstitution 1.4 mg) and subsequent washing, a second transport reaction was performed with RRYQNSTC^(FL488)^L (1 μM) for 10 min; **b**) or followed over time (**c**,**d**). In **b**, amount of Atto565-labelled peptide encapsulated in the lysosomes after first (1st) and second (2nd) transport is depicted, whereas in **c**,**d** show the amounts of Atto565-labelled (red), fluorescein-labelled (green) and total peptide (black) in the lumen of liposomes after different time points of the second transport process. Data are normalized to the first transport reaction of wt TAP and are presented as means±s.d. (*n*=3).

Membrane transport via alternating access can occur either uni- or bidirectionally. We therefore analysed the vectoriality by dual-colour import/export assays (Fig. 4a). Peptides were labelled by spectrally separated fluorophores, which affect neither their binding affinities nor transport kinetics^32^. First, the translocation of Atto565-labelled peptides was recorded. After washing, a second transport period was initiated under the same conditions by adding the fluorescein-labelled peptide. By parallel detection of both peptides, we observed that initially transported peptides were not retrotranslocated by wt TAP (Fig. 4b and Supplementary Fig. 7), as also established by observing the uptake of the differently labelled peptides over time during the second transport period (Fig. 4c). If retrotranslocation occurred, a decrease of Atto565-labelled peptide would be observed because re-uptake would be competed by the large excess of fluorescein peptide in the second transport period. On the contrary, the D674A/wt complex facilitated the retrotranslocation of initially translocated Atto565-labelled peptides at the expense of the fluorescein-labelled peptides, whereas the total lumenal peptide concentration remained constant (Fig. 4c,d). Counter-flow experiments with proteoliposomes prefilled with fluorescein-labelled peptides demonstrated that peptide influx and efflux by D674A/wt are not coupled but require ATP (Supplementary Fig. 7). In conclusion, wt TAP operates unidirectionally, even if the proteoliposomes contain saturating concentrations of peptide, whereas the D-loop mutant acts as a nucleotide-gated facilitator allowing for passive (downhill) flux in both directions.

### The D-loop influences the affinity for NBD dimerization

**Figure 5:**
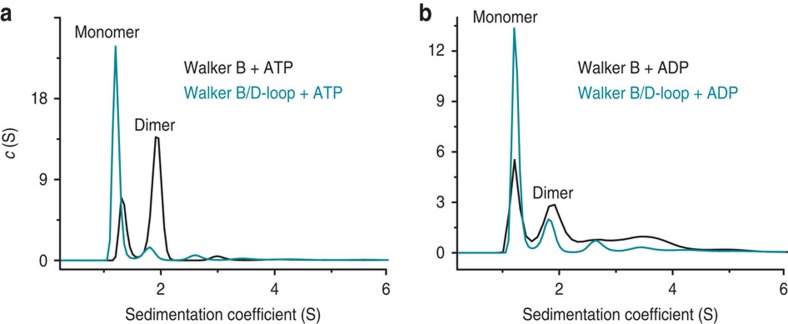
The D-loop D-to-A substitution greatly reduces ATP-driven NBD dimerization. (**a**,**b**) Sedimentation coefficient distributions derived from sedimentation velocity experiments. Distributions for Walker B D645N (black traces) and Walker B D645N/D-loop D651A (cyan traces) rat TAP1-NBD are shown in ATP (**a**) or ADP (**b**) containing buffer. Peaks are labelled ‘monomer’ and ‘dimer’ based on the calculated molecular weights (see Table 1).

To understand the D-loop substitution at a biochemical and structural level, we used a model system relying on homodimerization of the isolated rat TAP1-NBD^17^. This model system enables structural studies of NBD dimers as well as biophysical measurements of NBD dimerization affinities that are not accessible in full-length transporter systems. Importantly, the hydrolysis-deficient Walker B mutant (D645N) has a D-loop structure and interactions very similar to those seen in physiological NBD dimer structures^17^,^23^,^33^,^34^. We introduced the D-loop D651A mutation in this model system, which is equivalent to D674A in human TAP1, to study its effect on NBD dimerization, yielding two copies of the D-loop mutation in the homodimeric state. It should be noted that no heterodimeric ABC transporter or isolated NBDs in the fully closed state have been assembled and structurally analysed so far. Our homodimeric model system, although imperfect, does enable us to measure the impact of the D-loop mutation on dimerization. We determined the dissociation constant (*K*_d_) of the NBD dimer using analytical ultracentrifugation. As observed in sedimentation velocity experiments, sedimentation coefficient distributions displayed two populations, corresponding to NBD monomer and dimer (Fig. 5 and Table 1). The hydrolysis-deficient Walker B mutant was predominantly dimeric only in the presence of ATP, whereas in the presence of ADP the monomeric form was dominant. In contrast, the Walker B/D-loop double mutant is mostly monomeric regardless of the nucleotide present. *K*_d_s obtained from sedimentation equilibrium experiments (Table 1) confirm the sedimentation velocity results, and indicate ~8-fold decreased affinity of NBD dimerization for the double mutant TAP1-NBD in the presence of ATP, yielding a *K*_d_ similar to that in the presence of ADP. In summary, these results demonstrate that the rat TAP1-NBD dimerizes in an ATP-dependent manner, and that the D-loop substitution (present at both ATPase sites in this model system) essentially eliminates the increased affinity observed in the presence of ATP. In the context of the full-length transporter, a reduction of the affinity for NBD dimerization would not prevent, but would destabilize the outward-facing conformation.

### D-loop mutation subtly alters the NBD dimer structure

**Figure 6:**
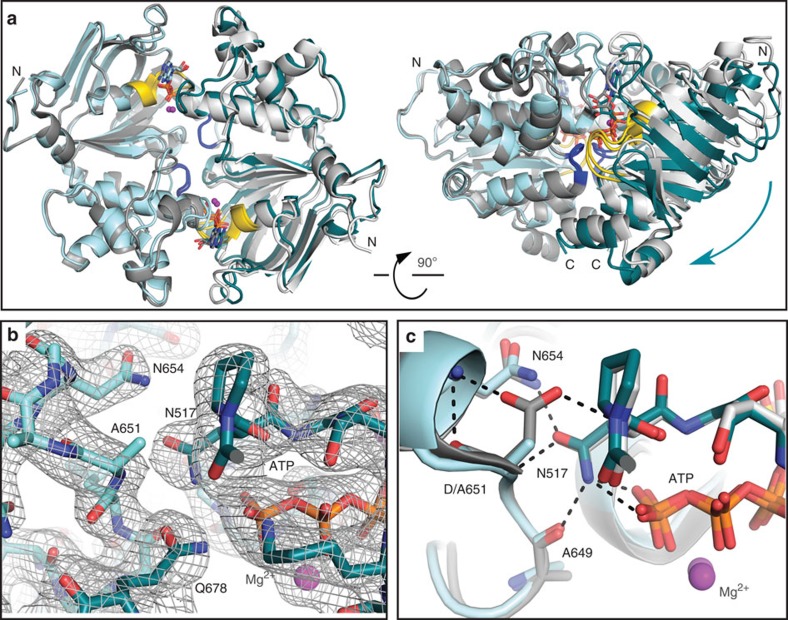
Stable NBD dimer formation and ATP hydrolysis serve as a checkpoint for unidirectional transport. (**a**) Overall alignment of dimers of rat TAP1-NBD Walker B D645N (grey, PDB 2IXE) and Walker B/D-loop D645N/D651A (cyan) mutants, viewed from the membrane plane (left) or side (right). The structures are superimposed using the protomer on the left to show the relative position of the protomer on the right, the cyan arrow showing the change. D-loops and Walker A motifs are blue and yellow, respectively. (**b**) 2F_o_−F_c_ electron density map contoured at 1σ showing the interface of the D-loop from one promoter (light cyan) and Walker A motif from the second promoter (dark cyan). (**c**) Detail of the dimer interface in **a** including the D-loop aspartate from one protomer and Walker A asparagine from the other protomer. Polar interactions are highlighted.

To visualize the structural changes caused by the D-loop substitution, we determined the crystal structure of Walker B/D-loop double mutant rat TAP1-NBD (Table 2). Interestingly, a dimer is observed through crystallographic twofold symmetry, revealing that although dimerization affinity is diminished, the double mutant can form the canonical NBD sandwich interface. Comparison with the previously determined Walker B mutant structure^17^, which contains an entire dimer in the asymmetric unit, reveals two significant differences. First, there is a global twist of the two protomers along the dimer interface (Fig. 6a and Supplementary Movie 1). Although the two ATPs and their binding sites are essentially unaffected by this structural re-organization, the C-terminal regions slide with respect to each other. Second, although the D-loop and interacting Walker A positions are essentially unaffected despite the loss of several hydrogen bonds (see below and Fig. 6b,c and Supplementary Fig. 8), the D-loop shows evidence of increased flexibility. A closer look at the TAP1-NBD structure reveals two key interactions for the D-loop aspartate. First, it interacts with the backbone of Walker A asparagine (N517) from the other protomer, a γ-phosphate sensor for the bound nucleotide^35^. Second, the D-loop aspartyl side chain caps the short helix that is C-terminal to it. In the D-loop mutant structure, the position and orientation of N517 is essentially unchanged, and it still interacts with the γ-phosphate, backbone of A649 and A651, and N654 side chain (Fig. 6c). Therefore, although the interactions through the D-loop aspartate side chain are lost due to mutation to alanine, the overall D-loop and Walker A positions are essentially unaffected. However, the B-factors for the D-loop and first turn of the helix that follows it (residues 648–655; 74.9 Å^2^) are significantly higher than the whole protomer average for the Walker B/D-loop double mutant (67.8 Å^2^), but not the Walker B mutant (24.2 Å^2^ for the D-loop versus a whole protomer average of 34.6 Å^2^), indicating that aspartate helps rigidify the D-loop region by acting as a helix cap. Both the global reorganization of the NBD dimer and the destabilization of the D-loop suggest that the D-to-A mutation in the TAP1 D-loop may impair communication between the ATPase sites in the NBDs and the peptide-binding site in the TMDs.

We note that our homodimeric TAP1 NBD structure represents only one state within the transport cycle, likely the fully closed NBD dimer state nearing ATP hydrolysis. Our structure also bears two copies of the D-loop mutation, unlike the heterodimeric D674A/wt complex that we observed behaves as a nucleotide-dependent facilitator. It is therefore possible that functional consequences of the D674A mutation stem at least in part from yet-to-be observed structural changes in the heterodimeric closed-NBD assembly and/or other steps in the transport cycle. Recent structures of a bacterial ABC exporter, TM287/288, provide some evidence for additional roles of the D-loop in NBD–NBD communication. Nearly identical structures of a nucleotide-free state and a state with one AMPPNP, bound to the degenerate ATPase site (root-mean-square deviation of 0.636 Å)^36^, both retain D-loop-mediated NBD–NBD contacts. Neither of these structures represents a fully closed-NBD dimer state, and these D-loop contacts are quite different from the ones we observed in our TAP1 NBD dimer.

## Discussion

To the best of our knowledge, our study establishes for the first time that TAP (i) indeed acts as an active transporter pumping peptides against a gradient and (ii) mechanistically operates as a molecular diode translocating peptides in a strict unidirectional mode. This formally excludes the possibility that TAP can retrotranslocate antigens to the cytosol, an important process in cross-presentation that is essential for the priming of naive cytotoxic T cells^37^. To define the vectoriality, the pathway from inward- to outward-facing conformation must differ from the reverse.

**Figure 7:**
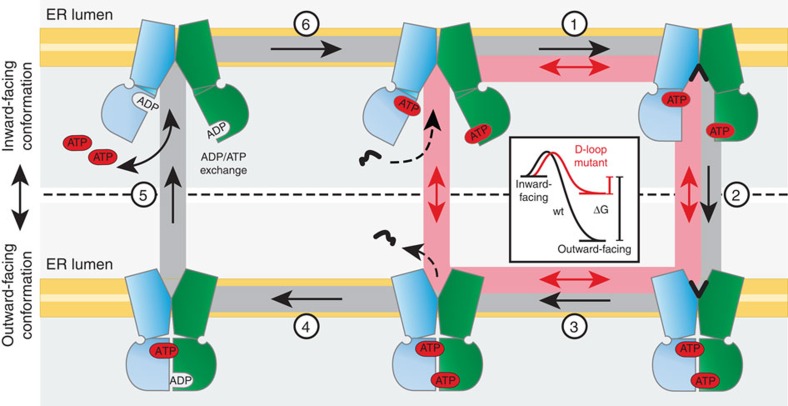
Model of the TAP transport cycle: stable NBD dimer formation and ATP hydrolysis serve as a checkpoint for unidirectional transport. In the resting state, the TAP NBDs are loaded with ATP. Peptide binding induces a conformation change (1), which triggers engagement of the NBDs and a conformational switch into the outward-facing ATP-bound state (2). Peptide release (3) triggers ATP hydrolysis (4), results in disengagement of the NBDs and restores the inward-facing conformation (5). The cycle is closed by an ADP/ATP exchange (6). The D-loop mutant can cycle without hydrolysis or peptide release in the ER lumen (red pathway), leading to a facilitator phenotype. The inset depicts the energetics of ATP-bound wt TAP (black) and the D674A/wt mutant (red). A high lumenal peptide concentration causes trans-inhibition of TAP by saturating an ER-lumenal-binding site, thereby inhibiting step 3.

Based on our findings we derive the following mechanistic model (Fig. 7): ATP and peptide, which can bind independently from each other^38^, trigger a conformational switch from the inward- to outward-facing conformation^39^. Peptide release into the ER triggers ATP hydrolysis, which is required for disassembly of the NBD dimer driving the outward-facing conformation back to the inward-facing one. Trans-inhibition takes place if peptide association to the ER-lumenal low-affinity binding site is faster than the conformational changes from the outward- into the inward-open state because of the high lumenal peptide concentration.

In the D674A/wt complex (red pathway), the loss of critical H-bonds and higher flexibility of the D-loop destabilize the NBD dimer and precludes ATP hydrolysis (Fig. 3a). Thus, NBD dissociation and the subsequent conformational change can occur independently from ATP hydrolysis and/or peptide release. Consequently, the inward- and outward-facing conformations can switch back and forth, leading to an uncoupled bidirectional flow of peptides along their gradient (Fig. 3). We propose that in wt TAP the dissociation of the dimer is driven by an intermediate, post-hydrolysis state that is necessarily incompatible with the dimer—much more so than ADP. The existence of this intermediate step (or rather, the need for the system to escape it) would impose vectoriality to the process. In the D-loop mutant, by contrast, association/dissociation is not directionally driven because ATP hydrolysis cannot occur. The D674A complex returns to the resting state by the reversal of the forward reaction and can therefore shuttle peptides in both directions. Therefore, the D-loop serves as one of likely several elements that contribute to vectoriality. Based on the principle of ‘microscopic reversibility’, the wt transport system should, in theory, run in the opposite direction at a very high lumenal-to-cytosolic peptide, high ADP and inorganic phosphate concentrations. However, we did not observe this in our experiments, suggesting that this pathway is kinetically disfavoured and TAP is arrested by trans-inhibition at high lumenal peptide concentrations. These findings present a prime example of kinetic versus thermodynamic control and of the mechanistic link between unidirectional, primary active ABC transporters and bidirectional, passive facilitators.

## Methods

### Functional assays

Baculovirus expression of TAP1 and TAP2 in *Sf*9 insect cells (Life Technologies), membrane preparation, solubilization, purification, reconstitution, peptide binding, peptide translocation and ATPase assays were performed as described^30^. D-loop substitutions were introduced in human TAP1 and TAP2 by standard cloning procedures. To analyse ATP binding, purified TAP was incubated with 8-azido-[α^32^P]ATP (1 μM) and MgCl_2_ (1 mM) for 5 min at 4 °C in standard buffer (20 mM HEPES, 140 mM NaCl, 15% glycerol, 0.05% digitonin, pH 7.4). After photo-crosslinking for 5 min, subunits of the TAP complex were analysed by SDS–PAGE (6%) and subsequent autoradiography was done. TAP2 was fused N-terminally to tapasin to allow for separation of both TAP subunits^40^. The antigen-processing pathway and MHC I surface expression was reconstituted by coexpression of the tapasin, HLA-B*4402 and TAP1/2 in *Sf*9 insect cells using baculovirus expression system^41^. The MHC I surface expression was analysed by flow cytometry using the conformation-specific anti-HLA-A/B/C antibody (W6/32) coupled to phycoerythrin (BioLegend). Monoclonal antibodies used for blots were anti-TAP1 mAb 148.3 (ref. 42), anti-TAP2 mAb 435.3 (ref. 43) and anti-tapasin mAb 7F6 (ref. 44).

### Peptide translocation in proteoliposomes

Purified TAP was reconstituted into liposomes of *Escherichia coli* polar lipids and 1,2-dioleoyl-*sn*-glycero-3-phosphocholine (70/30%) as described^30^. Proteoliposomes containing a lipid-to-TAP (w/w) ratio of 20 were incubated in transport buffer (20 mM HEPES, 140 mM NaCl, 3 mM MgCl_2_, 5% glycerol, pH 7.4) with indicated concentrations of the peptide RRYQNSTC^(FL488 or AT633)^L or RRYC^(FL488)^NSTEL^32^ in the presence or absence of ATP (3 mM) at 37 °C in a volume of 50 μl. Peptide transport was stopped by adding ice-cold stopping buffer (10 mM EDTA in phosphate-buffered saline) containing 100-fold molar excess of unlabelled peptide RRYQKSTEL to prevent peptide binding to TAP. Proteoliposomes were washed twice by centrifugation for 15 min at 125,000*g* at 4 °C. In the case of two consecutive transport periods, the first translocation period was performed with peptide RRYQNSTC^(AT565)^L. After intensive washing of proteoliposomes, a second transport period was conducted with peptide RRYQNSTC.^(FL448)^L. To determine the encapsulated volume of liposomes, 50 μM of 5(6)-carboxyfluorescein was added during the swelling of the lipid film and kept constant during the entire reconstitution procedure.

### Crystallization, data collection and refinement

Rat TAP1 NBDs were expressed and purified as before^17^. Briefly, the proteins were expressed in *E. coli* BL21(DE3), affinity-purified on Ni-NTA resin (Qiagen), eluting with an imidazole step gradient. Fractions containing the NBD were further purified on a Superdex200 column (GE Healthcare). All mutations were introduced by using QuikChange mutagenesis kit (primers for D645N: 5′-CTTATCTTGGACAATGCCACCAGTGC-3′ and 5′-CAGGGCACTGGTGGCATTGTCCAAGA-3′; primers for D651A: 5′-CCAGTGCCCTGGCTGCTGGCAACCAGCTACGGGTC-3′ and 5′-CTGGTTGCCAGCAGCCAGGGCACTGGTGGCATCGTC-3′) and confirmed by DNA sequencing. Rat TAP1 NBD D645N/D651A (15 mg ml^−1^) was crystallized at 4 °C in 1+1 μl sitting drops by vapour diffusion against reservoir solution (0.84 M Na_3_ citrate, 100 mM Tris, pH 8.0, 3.6 mM NiCl_2_). Crystals were frozen in reservoir solution plus 25% glycerol. Diffraction data were collected at Advanced Photon Source (APS) beamline 24-ID-C (NE-CAT, Argonne National Labs) and processed with X-ray Detector Software (XDS)^45^. Structure was determined by molecular replacement with PHASER^46^ using rat TAP1-NBD (D645N; 2IXE) with nucleotide removed as the search model. Model building and refinement were done with PHENIX^47^ and Coot^46^. Morph movie was made with the molmovdb.org server^48^. Figures were made in PyMOL (Schrödinger, LLC).

### Analytical ultracentrifugation

Protein samples (33 μM) dialysed against sample buffer (20 mM Tris, pH 8.0, 50 mM NaCl, 5 mM MgCl_2_) in the presence of the indicated nucleotide. Experiments were performed in an Optima XL-I analytical ultracentrifuge equipped with interference optics (Beckman Coulter) using an eight-hole An-50 Ti rotor (Beckman Coulter), and interference scans were acquired every 9 min. Buffer density, viscosity and partial specific volumes were estimated using SEDNTERP^49^. Sedimentation velocity experiments were run at 40,000 r.p.m. at 10 °C for 14 h in double sector cells with sapphire windows. Data were analysed according to continuous sedimentation coefficient distribution (c(S)) model in Sedfit^50^, to determine sedimentation coefficients for each sample. Sedimentation equilibrium experiments were performed at 12,000, 15,000 and 22,000 r.p.m. at 10 °C in six-sector cells using three different concentrations (3, 11 and 33 μM) of each sample or sample buffer containing the indicated nucleotide. After 24 h, samples reached equilibrium and ten interference spectra were recorded. Data were analysed according to the monomer-dimer self-association model in SedPhat^51^. The convergence of data fitting to a global minimum was verified using different methods including Simplex, Marquardt-Levenberg and Simulated Annealing.

### TAP1-NBD ATP hydrolysis

ATP hydrolysis assays were performed as described^41^, except TAP1-NBD protein samples were stripped of bound nucleotide by passing twice through a PD-10 column equilibrated with ATPase reaction buffer (150 mM potassium acetate, 10% glycerol, 1 mM dithiothreitol and 50 mM K-HEPES (pH 7.5)) and the A_260_/A_280_ ratio was verified to be <0.6. Data were collected on a FlexStation 3 (Molecular Devices).

### TAP1-NBD nucleotide binding

Nucleotide-stripped TAP1-NBD samples were re-concentrated to ~100 μM, and used to make a series of eight twofold dilutions in assay buffer (20 mM Tris, pH 8.0, 50 mM NaCl, 1 mM dithiothreitol) with 1 μM ATP-Bodipy (Invitrogen). A background control contained no protein. For competitive binding experiments, NBDs (25 μM final) were mixed with 1 μM ATP-Bodipy and a series of ten twofold dilutions of unlabelled nucleotide, with samples with no labelled nucleotide serving as background control. Fluorescence polarization was measured in FlexStation 3 with excitation at 488 nm. All samples were run two or three times in triplicates. Data were analysed with Origin (OriginLab Corp.). Binding affinities of unlabelled nucleotides (*K*_i_s) were obtained using *K*_i_=IC_50_/([*L*]/*K*_d_+1).

## Author contributions

N.G., R.A. and R.T. performed all experiments with the full-length TAP complex. A.S.V. and R.G. carried out all experiments with the NBDs of TAP. S.H. constructed the Tsn-TAP fusion construct. R.A., R.G. and R.T. designed the experiments, N.G., A.S.V., R.A., R.G. and R.T. analysed the data and wrote the paper.

## Additional information

**Accession codes:** The atomic coordinates and structure factors for the rat TAP1 NBD D645N/D651A structure have been deposited under accession code 4K8O in the Protein Data Bank.

**How to cite this article:** Grossmann, N. *et al.* Mechanistic determinants of the directionality and energetics of active export by a heterodimeric ABC transporter. *Nat. Commun.* 5:5419 doi: 10.1038/ncomms6419 (2014).

## Supplementary Material

Supplementary Information Supplementary Figures 1-8, Supplementary Tables 1-3 and Supplementary References.

Supplementary Movie 1 Morph of the Walker B D645N mutant TAP1-NBD dimer structure (2IXE) to the D645N/D651A double mutant TAP1-NBD dimer structure. The morph is animated first as view from the membrane, and then as viewed from the side. Conserved ABC transporter sequence motifs important for ATP binding and hydrolysis are colored: Walker A, yellow; Walker B, blue, D-loop, red; C-loop, green, Q-loop, pink, H-loop, orange.

## Figures and Tables

**Table 1:** Data analysis for sedimentation experiments.

**Mutant**	**Nucleotide**	**Sedimentation velocity**			**Sedimentation equilibrium**
		**Molecular weight in kDa**		**2nd/1st ratio**	***K***_**d**_ **for dimerization (μM)** *
		**1st Peak**	**2nd Peak**		
Walker B	ATP	28.0 (1.14)†	47.3 (2.97)†	2.61	22.4 (0.8)‡
Walker B/D-loop	ATP	28.6 (3.22)	50.7 (0.37)	0.11	170 (0.9)
Walker B	ADP	25.1 (1.20)	47.3 (0.82)	0.68	111 (0.9)
Walker B/D-loop	ADP	30.8 (1.97)	54.6 (0.52)	0.26	164 (1.0)

For the sedimentation velocity experiments, we list the molecular weights from best fits of the distributions. The ratio of integration of the two peaks is a measure of the ratio of dimer to monomer populations. For the sedimentation equilibrium experiments, we list the TAP1-NBD dimerization constants obtained.

^*^Ninety-five percent confidence intervals are as follows: Walker B D645N+ATP or ADP, 22.1–23.21 or 56–187, respectively; and Walker B D645N/D-loop D651A+ATP or ADP, 103–118 or 156–174, respectively.

^†^Numbers in parentheses correspond to the integration of each peak shown in Fig. 5.

^‡^Numbers in parentheses are the global *χ*^2^ values.

**Table 2:** Data collection and refinement statistics.

*Data collection*	
Space group	C222_1_
	
*Cell dimensions*	
*a*, *b*, *c* (Å)	54.26, 123.98, 79.91
*α*, *β*, *γ* (°)	90, 90, 90
Resolution (Å)	48.98–2.65 (2.74–2.65)*
*R*_*meas*_	0.139 (2.89)
*I*/σ*I*	7.23 (0.58)
Completeness (%)	95.9 (97.5)
Redundancy	3.0 (2.9)
CC(1/2) (%)	99.4 (20.5)
	
*Refinement*	
Resolution (Å)	48.98–2.65
No. of reflections (test set)	7039 (782)
*R*_work_/*R*_free_	24.71/27.02
	
*No. of atoms*	
Protein	1,930
Ligand/ion	46
Water	20
	
*B-factors*	
Protein	67.7
Ligand/ion	56.2
Water	52.7
	
*Root-mean-square deviations*	
Bond lengths (Å)	0.003
Bond angles (°)	0.717
PDB code	4K8O

PDB, Protein Data Bank.

^*^Values in parentheses are for highest-resolution shell.
