# Supplementary material for: Mechanistic determinants of the directionality and energetics of active export by a heterodimeric ABC transporter
Source: Nat Commun. 2014 Nov 7;5:5419. doi: 10.1038/ncomms6419 (PMC4242082; doi:10.1038/ncomms6419)
Supplement: Supplementary Information — Supplementary Figures 1-8, Supplementary Tables 1-3 and Supplementary References. [file ncomms6419-s1.pdf]

Supplementary Figures

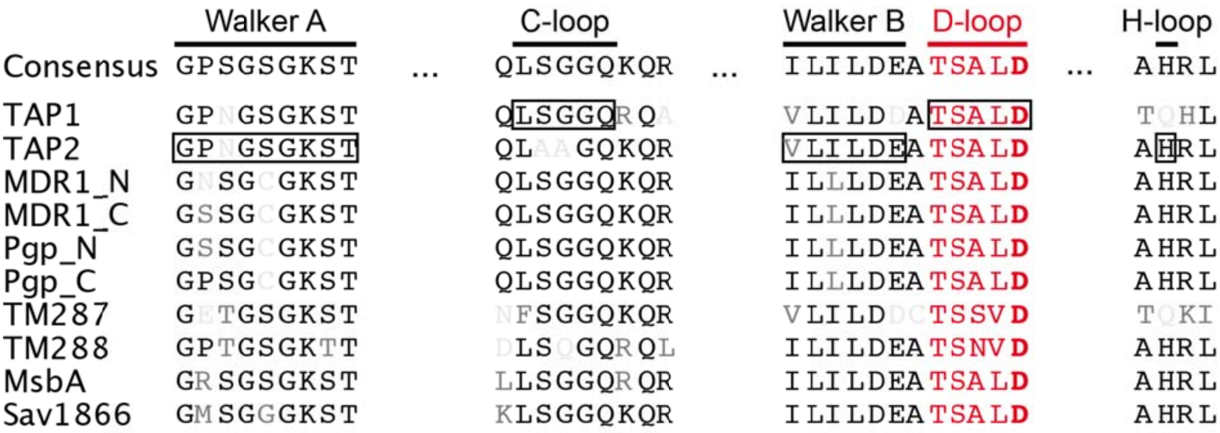

Supplementary Figure 1 | Consensus motifs involved in ATP binding and hydrolysis. Multiple sequence alignment of ABC transporters (human TAP1/2 (Q03518/9) with a sampling of other hetero- and homodimeric ABC transporters, human P-gp (MDR1, P08183); *Caenorhabditis elegans* P-gp (P34712*); Thermotoga maritima* ORF 0287/8 (GI:15643056/7); *Salmonella typhimurium* MsbA (P63359); *Staphylococcus aureus* Sav1866 (Q99T13)), was performed using ClustalW2. Sequence motifs are highlighted. The D-loop and its conserved aspartate (bold) are colored in red. Motifs that correspond to the consensus active site II are highlighted by black boxes. Conserved and non-conserved substitutions are marked in light and dark grey, respectively.

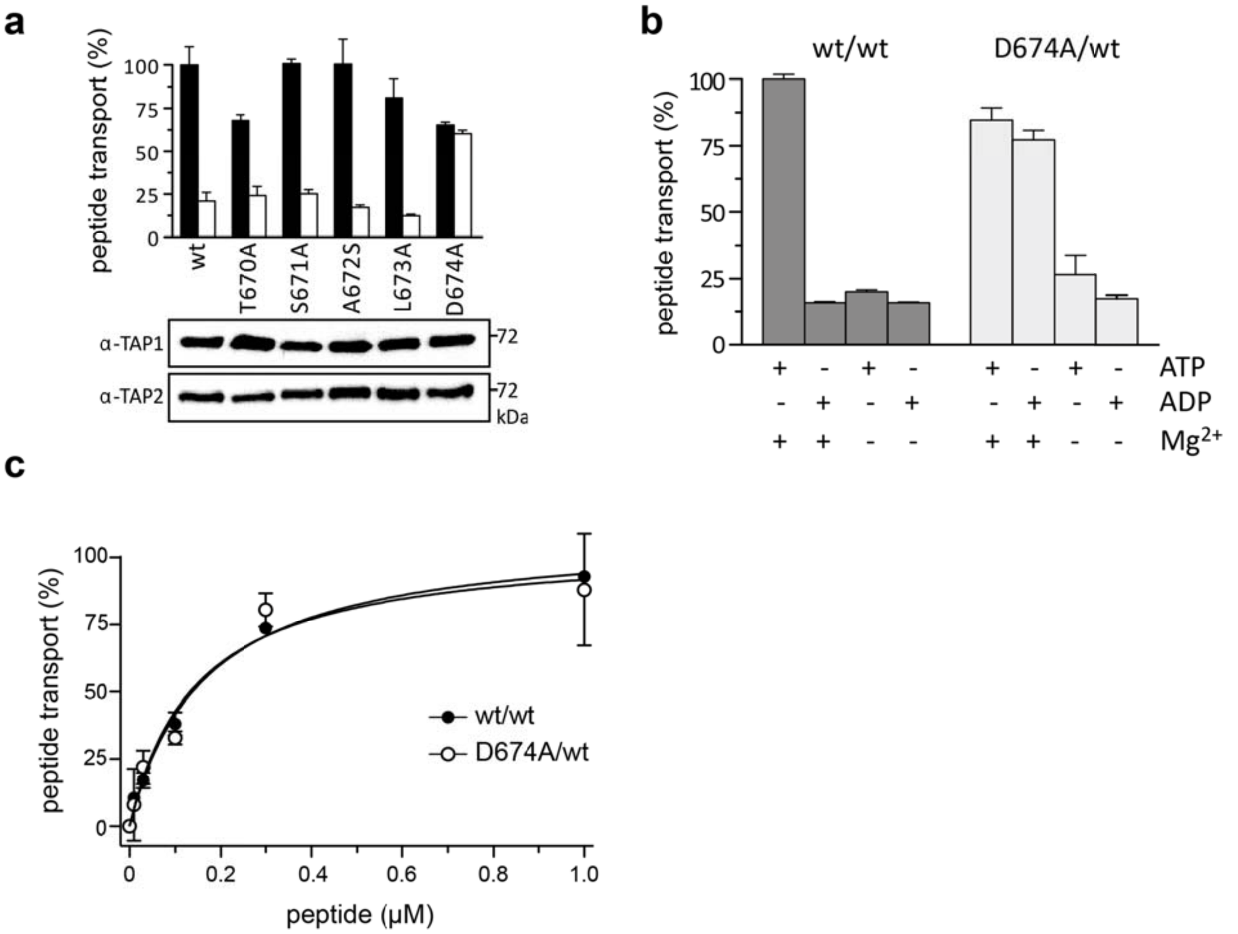

Supplementary Figure 2 | Peptide transport of the wt TAP and D-loop variants. (**a**) Transport of fluorescein-labeled peptide RRYQNSTC$^{(FL488)}$L (1 μM) by microsomal wt TAP and D-loop mutants was analyzed in the presence of ATP (black) or ADP (white) (3 mM) for 3 min. Transported peptides were quantified fluorometrically ($\lambda_{ex/em}$ 485/520 nm). Equal expression levels of TAP1 and TAP2 were confirmed by immunoblotting. (**b**) Transport of RRYC$^{(FL488)}$KSTEL (1 μM) by reconstituted wt TAP and D674A/wt (at an initial lipid-to-TAP w/w ratio of 20 and initial TAP amount of 0.7 μg)) was studied with ATP or ADP (3 mM each) in the presence or absence of $MgCl_2$ (5 mM) for 10 min. (**c**) The peptide dependence of transport was studied in the presence of ATP (3 mM). The corresponding K_m values are summarized in Supplementary Table 3. All data are presented as means ± SD (n = 3).

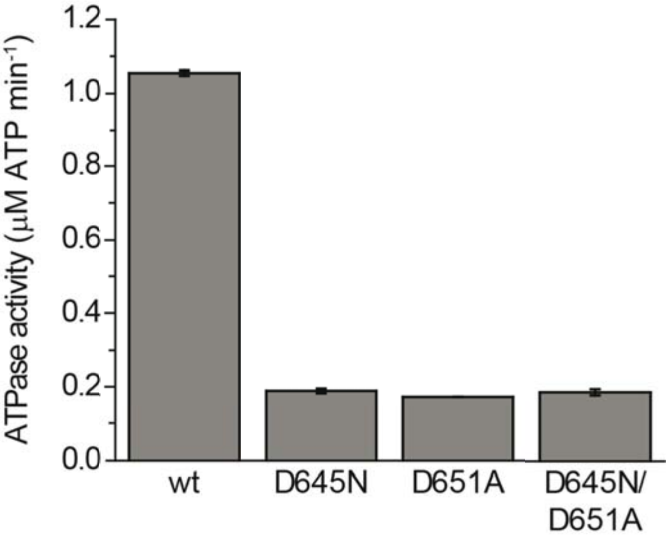

Supplementary Figure 3 | Mutation of the D-loop aspartate to alanine greatly reduces basal ATPase activity of the isolated rat TAP1-NBD. Basal ATPase activity of the isolated rat wt TAP1-NBD, or the D-loop (D651A), hydrolysis-deficient Walker B (D645N) mutant, or double mutant (D645N/D651A), measured using a colorimetric NADH-coupled ATPase assay. All proteins were at 15 μM. As previously observed[1], the classical "hydrolysis-deficient" Walker B D645N mutant showed nearly an order of magnitude lower ATPase activity compared to wt rat TAP1-NBD. The D-loop D651A mutant and the double mutant similarly showed little or no ATPase activity. Therefore, although the D-loop Asp651 does not directly interact with the bound nucleotide, the D-loop mutation is sufficient to impair basal ATP hydrolysis. ATPase activity at 1 mM ATP is plotted as means ± SD.

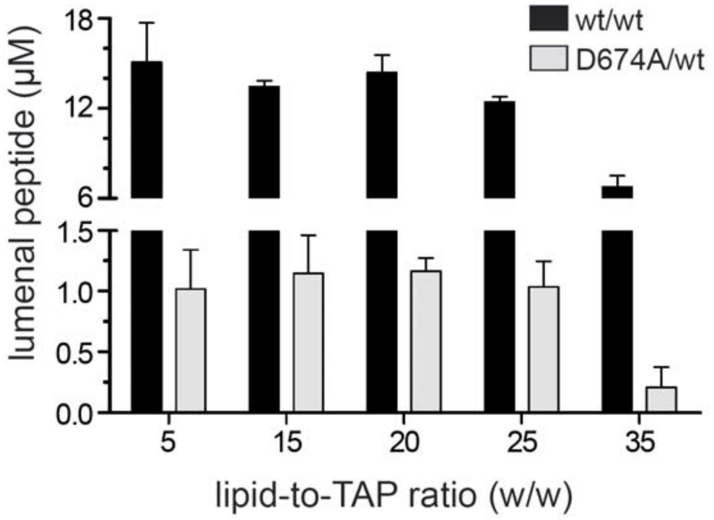

Supplementary Figure 4 | Peptide transport as function of the initial lipid-to-TAP ratio used in reconstitution. wt TAP or the D674A/wt complex (0.7 µg each) were reconstituted at increasing lipid-to-protein ratios and peptide transport was analyzed in the presence of RRYC$^{(AT633)}$KSTEL (1 µM) and ATP (3 mM) after 1 h. To determine the concentration of lumenal peptides after translocation, the amount of ATP specific transported peptides ($\lambda_{ex/em}$ 630/645 nm) was normalized to the internal volume of the liposomes determined by 5(6)-carboxyfluorescein ($\lambda_{ex/em}$ 490/517 nm) encapsulated in the proteoliposomes during reconstitution. Peptide accumulation was the same between a ratio of 5 to 25. This indicates that each liposome has an active TAP complex (as shown in Supplementary Figure 5) and therefore maximal filling is not influenced by low reconstitution efficiency. By lowering the lipid-to-TAP ratio a critical threshold is reached below which less than one active TAP is reconstituted in the correct orientation. Data are presented as means ± SD (n = 3).

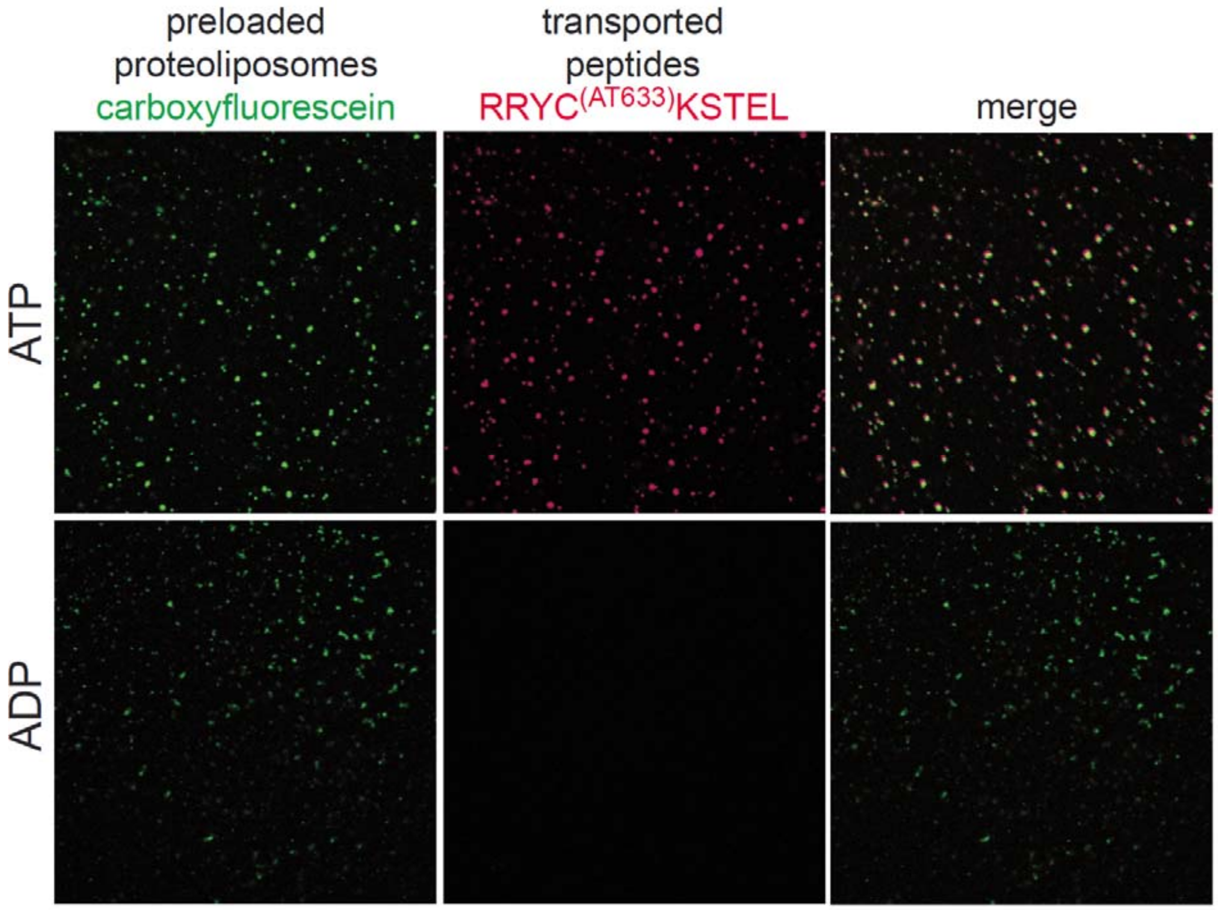

Supplementary Figure 5 | Single vesicle based transport assays establish that each liposome contains at least one active transport complex in correct orientation. Purified wt TAP (0.7 µg) was reconstituted at an initial lipid-to-protein ratio of 20:1 (w/w). Proteoliposomes containing encapsulated 5(6)-carboxyfluorescein (50 µM). The transport of RRYC$^{(AT633)}$KSTEL (50 µM) was followed in the presence of ATP or ADP (3 mM each) for 30 min. After washing, the proteoliposomes and transported peptides were visualized by confocal laser scanning microscopy. As derived from the merged images nearly all proteoliposomes contained functional TAP, whereas no peptide accumulation was detected in the absence of ATP. This demonstrates that the critical threshold of lumenal peptides is not biased by a fraction of liposomes devoid of functional TAP. The vesicle diameter of 200 nm has been determined by nanoparticle tracking[2].

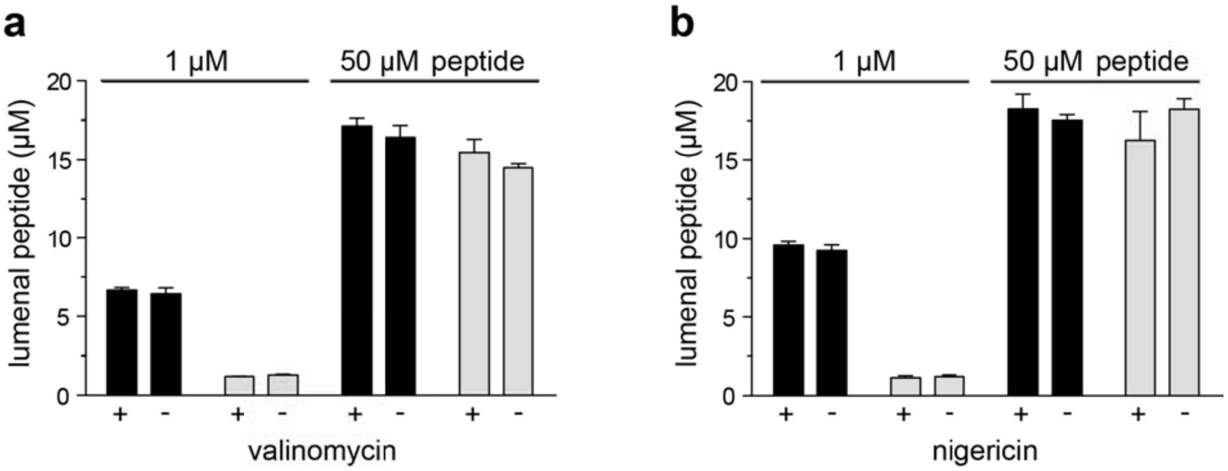

Supplementary Figure 6 | Peptide transport by TAP is not affected by proton and ion gradients. Proteoliposomes containing wt TAP (black) or D674A/wt (grey; initial amount of 0.7 µg each)were incubated with the peptide RRYC$^{(AT633)}$KSTEL (1 µM or 50 µM) and ATP (3 mM) for 1 h in the presence or absence of the K^+ specific ionophore valinomycin (**a**) or the K^+ and H^+ specific ionophore nigericin (**b**, 1 µM each). For transport studies with valinomycin, potassium phosphate (20 mM) was present inside and outside the proteoliposomes. The amount of transported peptides ($\lambda_{ex/em}$ 630/645 nm) was normalized to the internal volume of the liposomes determined by 5(6)-carboxyfluorescein ($\lambda_{ex/em}$ 490/517 nm) encapsulated during reconstitution. Data are presented as means ± SD (n = 3).

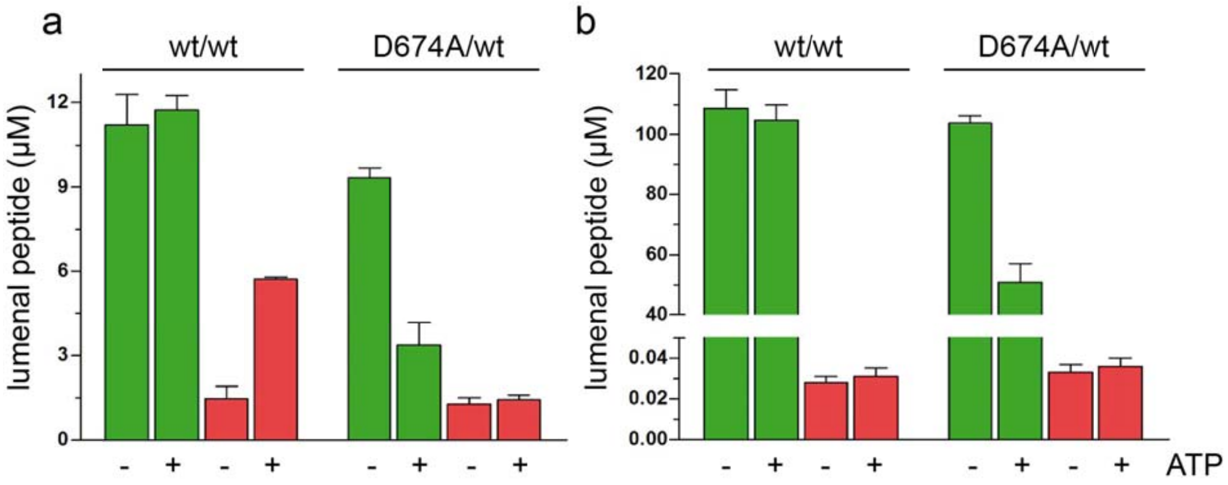

Supplementary Figure 7 | Counter-flow experiments with prefilled proteoliposomes show that peptide influx and efflux by D674A/wt are not coupled but require the presence of ATP. Transport assay with proteoliposomes containing wt TAP or D674A/wt (initial amount of 1.9 µg each) prefilled with RRYC$^{(FL488)}$KSTEL (10 µM (**a**) or 100 µM (**b**); green bars) was performed with RRYC$^{(AT633)}$KSTEL (1 µM, red bars) for 10 min in the presence or absence of ATP (3 mM). Depicted is the corresponding lumenal peptide concentration after the counter-flow experiment. D674/wt complex shows an ATP dependent passive efflux of peptides, whereas wt TAP cannot retrotranslocate peptides. Importantly, the D674A/wt complex does not accumulate peptide above the concentration of the peptide added to the outside of liposomes excluding a coupling of import and export. In contrast, for wt TAP peptide can be enriched up to a total concentration of 16 µM (ca. 10 µM prefilled RRYC$^{(FL488)}$KSTEL plus 6 µM transported RRYC$^{(AT633)}$KSTEL). Further transport is inhibited by trans-inhibition. For liposomes filled with 100 µM of peptide no peptide import is detectable because of trans-inhibition. Data are presented as means ± SD (n = 3).

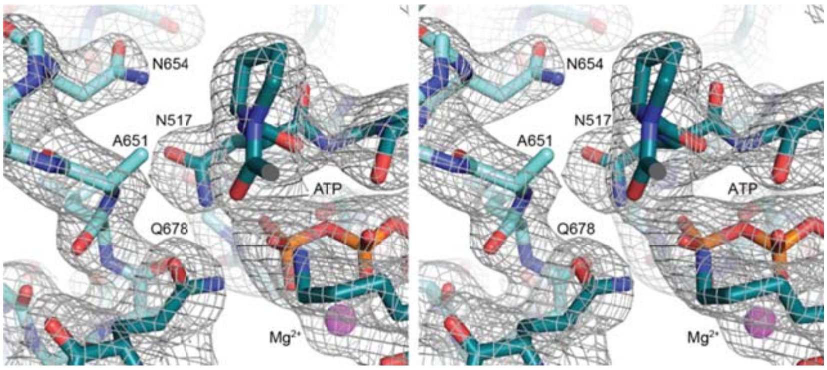

Supplementary Figure 8: Stereo representation of the final $2F_o$-F_c electron density map for the D-loop. The map is contoured at 1σ, showing the interface of the D-loop from one protomer (light cyan) and Walker A motif from the second protomer (dark cyan). Residue A651 was mutated from aspartate in this structure, in comparison to the previously determined structure (2IXE)[1] used for molecular replacement.

Supplementary Tables

Supplementary Table 1 Peptide and ATP binding with TAP containing membranes were investigated by filter binding and ATP-agarose competition assays, respectively.

TAP1/TAP2 complex	Peptide binding* K_d (μM)	ATP binding IC_{50} (μM)
wt/wt	0.4 ± 0.1	10 ± 1
T670A/wt	0.6 ± 0.2	50 ± 13
S671A/wt	0.5 ± 0.1	8 ± 1
A672S/wt	0.6 ± 0.1	16 ± 1
L673A/wt	0.5 ± 0.1	25 ± 5
D674A/wt	0.6 ± 0.1	40 ± 5
wt/D638A	0.3 ± 0.1	72 ± 18
D674A/D638A	0.4 ± 0.1	37 ± 6

*RR[^{125}I]YQKSTEL; ± SD (n = 6)

Supplementary Table 2 Binding affinities of the rat wt and D-loop D651A mutant TAP1-NBD proteins to a panel of nucleotides. Binding of ATP-Bodipy (1 μM) to rat NBDs quantified by fluorescence polarization assay (K_ds). IC_{50} values calculated from competitive binding experiments with unlabeled ATP or ADP. Binding affinities of unlabeled nucleotides (K_is) obtained using K_i=IC_{50}/([L]/K_d +1).

	**K_d (μM) (ATP-Bodipy)**	**IC_{50} (μM)**		**K_i (μM)**	
		**ATP**	**ADP**	**ATP**	**ADP**
**WT**	6.1 ± 1.7	139 ± 30	69 ± 14	119 ± 13	59 ± 2
**D-loop D651A**	5.9 ± 0.6	173 ± 29	69 ± 19	148 ± 9	59 ± 3

Supplementary Table 3 Peptide transport kinetics of the reconstituted wt/wt and D674A/wt TAP complex.

TAP1/TAP2 complex	$K_{m,ATP}$ (μM)	$K_{m,peptide*}$ (nM)
wt/wt	99 ± 24	63 ± 26
D674A/wt	123 ± 18	86 ± 36

*RRYC$^{(FL488)}$KSTEL

Supplementary References:

1. Procko E, Ferrin-O'Connell I, Ng SL, Gaudet R. Distinct structural and functional properties of the ATPase sites in an asymmetric ABC transporter. *Mol Cell* **24**, 51-62 (2006).
2. Urban M, *et al.* Highly parallel transport recordings on a membrane-on-nanopore chip at single molecule resolution. *Nano Lett* **14**, 1674-1680 (2014).
